# Antimicrobial Activity of Morphology-Controlled Cu_2_O Nanoparticles: Oxidation Stability under Humid and Thermal Conditions

**DOI:** 10.3390/ma17010261

**Published:** 2024-01-04

**Authors:** Jeong Yeon Park, Siwoo Lee, Yangdo Kim, Young Bok Ryu

**Affiliations:** 1 Green Materials and Process R&D Group, Korea Institute of Industrial Technology, Ulsan 44413, Republic of Korea; jyeon00@kitech.re.kr (J.Y.P.); lissue88@kitech.re.kr (S.L.); 2 Department of Materials Science and Engineering, Pusan National University, Busan 46241, Republic of Korea

**Keywords:** cuprous oxide, nanoparticle, morphology, antimicrobial activity, oxidation stability

## Abstract

Metal oxides can be used as antimicrobial agents, especially since they can be fabricated into various forms such as films, masks, and filters. In particular, the durability of antimicrobial agents and the duration of their antimicrobial activity are important factors that determine their suitability for a specific purpose. These factors are related to the morphology and size of particles. The metal oxide Cu_2_O is often oxidized to CuO in various conditions, which reduces its antimicrobial activity. This study focused on the oxidation of nanoparticles of Cu_2_O with three morphologies, namely, spherical, octahedral, and cubic morphologies, in excessively humid and excessive-thermal environments for a specific duration and the antimicrobial activity of the NPs. Cu_2_O nanoparticles were prepared using the chemical reduction method, and their morphology could be varied by adjusting the molar ratio of OH^−^ to Cu^2+^ and changing the reducing agent. It was found that cubic Cu_2_O was the most stable against oxidation and had the smallest reduction in antimicrobial activity. This study examined the antimicrobial activity and the oxidation stability of Cu_2_O NPs with different morphologies but similar particle sizes.

## 1. Introduction

The emergence of new strains of bacteria, fungi, and viruses poses a significant threat to human health. Microorganisms grow in warm and damp environments, which are widespread in many areas. Many techniques have been developed to kill pathogenic microorganisms, and the use of antimicrobial materials is one of them [1]. Various metals and metal oxides, including Ag [2], Cu [3], and ZnO [4], have been investigated for their antimicrobial activity against a wide variety of microorganisms. These metal and metal oxides nanoparticles (NPs) as antimicrobial agents offer the advantage of a long-term efficacy due to their solid characteristics. Furthermore, these nanostructures can exhibit antimicrobial properties through various attachment methods tailored to specific application areas.

Copper (Cu) is cost-effective and exhibits wide-spectrum antimicrobial activity, including against Gram-positive and Gram-negative microorganisms [5]. Previous studies have shown that metals and metal oxides employ mechanisms for microbial eradication, which are the release of copper ion with the production of reactive oxygen species (ROSs), subsequently leading to microbial death [6,7]. Wu et al. reported the variation in copper ion release rate based on the morphology of Cu_2_O [8]. Cu-based materials with antimicrobial activity are used in various fields, including the agricultural, industrial, and transportation fields as well as the field of drinking water treatment [9,10,11,12,13,14].

Cu-based materials can exist in three different forms, depending on their oxidation state: Cu (0), Cu_2_O (+1), and CuO (+2). Copper (Cu) has major drawbacks, such as being more rapidly oxidized upon exposure to air than Copper Oxides (Cu_x_O). Upon being oxidized, Cu transforms into CuO, which has lower antimicrobial activity. Similarly, Cu_2_O is oxidized to CuO in air over time. Several studies have demonstrated that Cu_2_O exhibits higher antimicrobial activity than CuO since it can release more toxic Cu^+^ ions [15,16,17]. Hence, Cu_2_O NPs have attracted considerable attention because of their characteristics.

The antimicrobial activity of NPs is related to their morphology, size, and surface area [18]. Several studies have shown that the morphology of Cu_2_O particles determines their physical and chemical properties [19,20,21,22]. Pang et al. investigated the antibacterial activity of Cu_2_O particles with various morphologies, such as octahedral, cubic, rodlike, and corner-truncated [23], and Lee et al. reported that cubic Cu_2_O showed higher antibacterial activity than octahedral Cu_2_O against *Escherichia coli* [24]. However, most studies have neglected the effect of particle size and have simply attributed antimicrobial activity to the morphology of particles. Even among studies pertaining to the morphology of Cu_2_O NPs, few studies have examined the effect of morphology on antimicrobial activity.

The characteristics (size, morphology, composition, oxidation state, etc.) of Cu_2_O particles could be changed by their environmental conditions. For example, upon exposure to moist air or high temperatures, Cu_2_O is oxidized to CuO. Many studies have investigated the antimicrobial activity of Cu_2_O. However, few studies have examined the long-lasting nature of Cu_2_O in oxidative environments. The use of antimicrobial agents is common in environments conducive to microorganisms’ growth. Such environments typically involve specific moisture conditions and temperatures [25]. For instance, the relative humidity (RH) required for supporting fungal growth on wood, gypsum, and ceramics has been reported to be above 75% in the temperature range of 20–25 °C [26]. For the fabrication of filters by using Cu_2_O NPs, several studies have reported controlling the air temperature at 250 °C and maintaining the polymer melt temperature at around 230–260 °C [27,28]. In particular, it is necessary to determine whether the antimicrobial activity of Cu_2_O is affected by factors such as temperature, humidity, and interactions with substance, which involves oxidation.

In this study, we investigated the oxidation stability and antimicrobial activity of Cu_2_O NPs with three morphologies: spherical, octahedral, and cubic morphologies. We modified a previously developed chemical reduction method to prepare Cu_2_O NPs at room temperature. We examined whether the antimicrobial activity changed after exposure to oxidative conditions, including humid conditions (85 ± 5%, 20 ± 5 °C) and melt-blown process (250 °C, 2 h) conditions. This study provides information that can help determine whether Cu_2_O would be suitable for a specific purpose.

## 2. Materials and Methods

### 2.1. Materials

Copper(II) nitrate trihydrate (Cu(NO_3_)_2_·3H_2_O, 99–104%) and hydrazine hydrate (N_2_H_4_·xH_2_O, 50–60%) were purchased from Sigma-Aldrich (Steinheim, Germany), and sodium hydroxide beads (NaOH, 97%) and L(+)-ascorbic acid (C_6_H_8_O_6_, 99%) were purchased from Daejung (Siheung, Republic of Korea). Deionized (DI) water with a resistivity of 18.2 MΩ·cm was prepared using a water purification system (aquapuri 5 series, Young-In, Anyang, Republic of Korea).

### 2.2. Preparation of Cu_2_O with Three Morphologies

**Figure 1:**
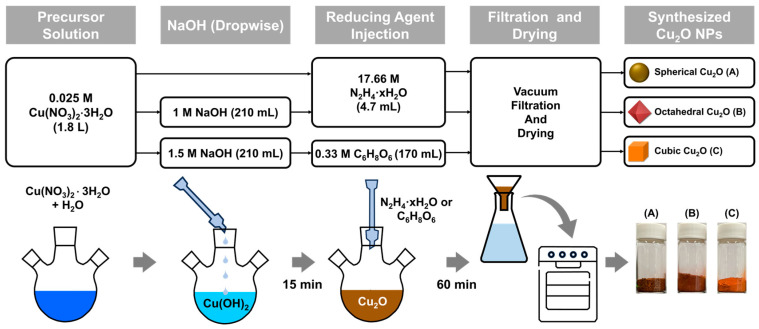
Flowchart and a schematic of the synthesis process of Cu_2_O NPs (A: Spherical Cu_2_O, B: Octahedral Cu_2_O, C: Cubic Cu_2_O).

Cu_2_O NPs with three morphologies were synthesized using a chemical reduction method in Figure 1. The precursor solution that comprised Cu(NO_3_)_2_·3H_2_O (0.025 M) in 1.8 L of DI water was stirred at 300 rpm using an impeller at 20 ± 3 °C (Supplementary Materials, Figures S1 and S2). After 15 min of stirring, 4.7 mL of N_2_H_4_·xH_2_O (17.66 M), which was used as a reducing agent, was injected (for spherical Cu_2_O). Before the addition of the reducing agent, 210 mL of NaOH solution (1 M for octahedral Cu_2_O and 1.5 M for cubic Cu_2_O) was added dropwise to the precursor solution at a rate of 5 mL/min during stirring by using a peristaltic pump. After 15 min of stirring, 4.7 mL of N_2_H_4_·xH_2_O (17.66 M; for octahedral Cu_2_O) or 170 mL of C_6_H_8_O_6_ (0.33 M; for cubic Cu_2_O), which was used as a reducing agent, was injected. The prepared mixture was stirred for 60 min. Cu_2_O particles were obtained through vacuum filtration, and they were washed with DI water several times to remove impurities. The particles were then placed in a vacuum oven pump at 30 °C for 6 h.

### 2.3. Characterization

The morphology, size, and structure of Cu_2_O NPs with different morphologies were investigated using cold field emission scanning electron microscopy (SEM, SU820, Hitachi, Tokyo, Japan) at an accelerating voltage of 10 kV and high-resolution transmission electron microscopy (HRTEM, Talos F200X, FEI Company, Hillsboro, OR, USA) at 200 kV. The ratio value of facets was determined through peak analysis of data obtained using X-ray diffraction (XRD, PANalytical Xpert 3 Powder, Malvern, Worcestershire, UK) with Cu Kα radiation of 1.5406 Å. The 2θ scanning range was 10°–90°, and the scan rate was 0.22°/s. The physical properties of the prepared samples were studied on the basis of N_2_ adsorption–desorption at 77 K by using an ASAP 2020 instrument (Micromeritics, Norcross, GA, USA). The surface area was calculated using the Brunauer–Emmett–Teller (BET) method in the relative pressure (*P*/*P*_0_) range of 0.05–0.3. Each sample was degassed under vacuum (<10 mm Hg) at 100 °C for 4 h before N_2_ physisorption. The thermal stability of Cu_2_O NPs was determined by Thermogravimetric analysis (TGA; TGA/DSC 3+, Mettler Toledo, Zurich, Switzerland). For the surface analysis of the Cu_2_O NPs, X-ray photoelectron spectroscopy (XPS, K-Alpha^+^, Thermo Fisher Scientific, Waltham, MA, USA) was performed with Al Kα (1486.6 eV) radiation. Core-level shifts were analyzed to extract information on the oxidation states of Cu and O atoms. Furthermore, for XPS analyses, internal carbon was used for charge calibration of the spectra; C 1s scan showed various peaks near the binding energy of 284.8 eV.

### 2.4. Oxidation Stability Test

Cu_2_O NPs were exposed for one, two, four, and eight weeks to humid environments. These environments were similar to those where filters containing Cu_2_O NPs are generally used. Each sample comprised 0.3 g of Cu_2_O powder in a 10 mL lidless vial. Subsequently, SEM, XRD, XPS, and an antimicrobial test were performed. The relative humidity (RH) and temperature of the chamber (JEIO TECH, Temperature & Humidity Chamber TH-KE-100, Republic of Korea) were regulated at 85% and 20 °C. The thermal stability of Cu_2_O was assessed using TGA and assuming melt blowing conditions. After performing TGA, Cu_2_O samples were confirmed with a focus on changes in XRD patterns. Ambient air gas was used as the carrier gas, and its flow rate was 10 mL/min. Cu_2_O powder weighing 0.3 mg (±1.0 mg) was taken in a ceramic sample pan, and the pan was heated from ambient temperature to 250 °C at a rate of 10 °C/min and maintained at the temperature for 2 h.

### 2.5. Antimicrobial Assays

The antimicrobial activity of each Cu_2_O sample was ascertained from data on the number of colony-forming units (CFUs). *Staphylococcus aureus* was collected using pipette swabs (3M^TM^ Pipette Swab Plus, USA), which were immediately immersed in 10 mL of pipette swab solution. The collected samples were serially diluted from 1 to 10^5^ by dispensing a 1 mL aliquot of the solution onto 9 mL of saline containing 0.85% sodium chloride. The dilutions of the control group were determined to be equivalent to or lower than 150 CFU/mL. Next, 1 mL of each dilution was plated onto a single 3M^TM^ Petrifilm Aerobic Count Plate, and the plate was incubated at 37 ± 1 °C for 48 h. One of the colonies in the plate was transferred to 9 mL of buffered peptone water (BPW) and cultured in an incubator at 37 ± 1 °C for 24 h. The as-prepared BPW was diluted 1–10^5^ times, and the control group of microbial solution was determined. In the experimental group, 30 µg/mL of Cu_2_O NPs was prepared and mixed with the control group solution in a volume ratio 1:1. The mixture was left undisturbed for an hour and subsequently cultured. After 48 h of incubation, counts on the plate were compared.

## 3. Results and Discussion

### 3.1. Structure and Morphology

**Figure 2:**
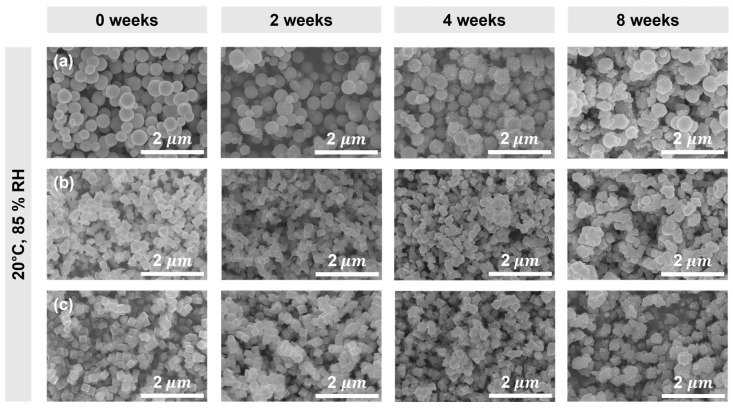
SEM images of Cu_2_O NPs with (**a**) spherical, (**b**) octahedral, and (**c**) cubic morphologies showing the effect of zero-, two-, four-, and eight-week exposure to humid conditions (temperature of 20 ± 5 °C and RH of 85 ± 5%).

Cu_2_O NPs with spherical, octahedral, and cubic morphologies were prepared through the chemical reduction method. The SEM images of as-prepared Cu_2_O NPs in Figure 2 show the morphological changes that occurred during the exposure of the samples in humid conditions. Spherical Cu_2_O was synthesized using a strong reducing agent, N_2_H_4_·xH_2_O, without the addition of NaOH. To stabilize their high-energy surfaces, the Cu_2_O seed NPs tended to aggregate and form large particles sizes of 100–615 nm [29]. Octahedral Cu_2_O was prepared by the reduction of Cu^2+^ ions with N_2_H_4_·xH_2_O in the presence of NaOH. Upon the addition of NaOH solution to copper salt solution, deep blue Cu(OH)_2_ precipitated. With 1 M NaOH, uniform octahedral NPs with well-defined edges and with particle sizes of about 250 nm were produced [30]. This preferential growth along the <100> direction resulted from the higher adsorption of OH^−^ on the (111) crystal facet compared with the other facets. As the concentration of NaOH increased, the number of OH^−^ ions available for adsorption also increased, leading to a larger number of octahedrons with (111) facets being formed [31].

**Figure 3:**
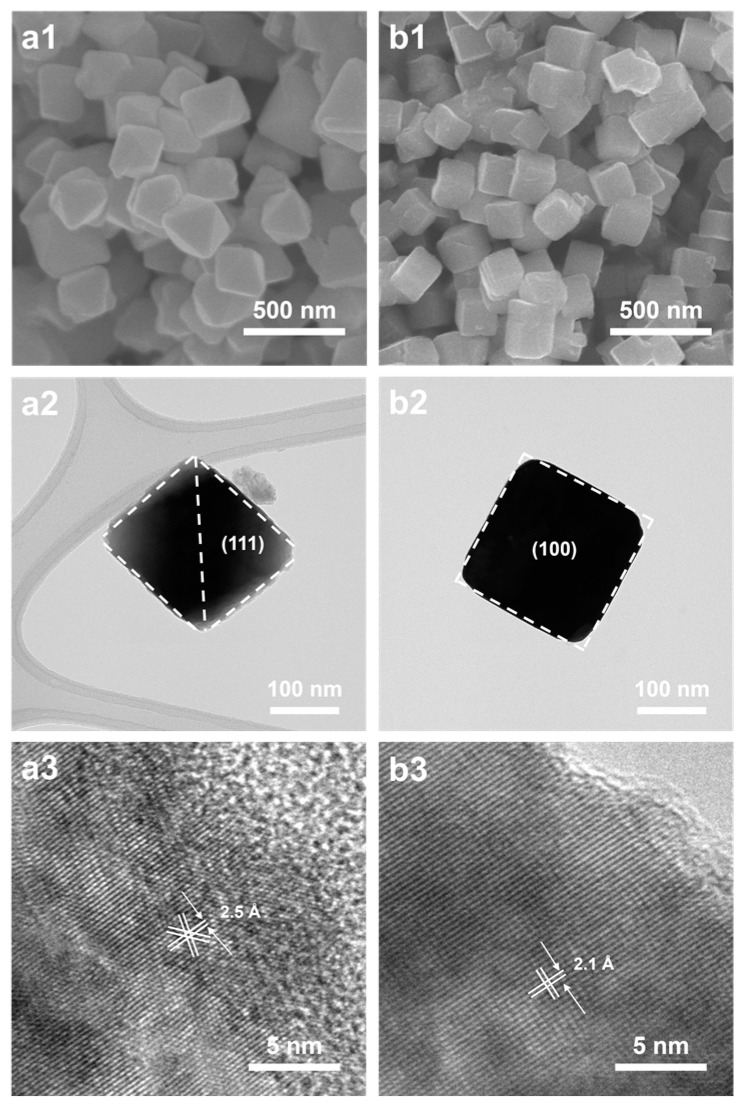
SEM images of as-synthesized (**a1**) octahedral and (**b1**) cubic Cu_2_O; HRTEM images of as-synthesized (**a2**,**a3**) octahedral and (**b2**,**b3**) cubic Cu_2_O.

The preferential adsorption restricted the growth rate of other crystal faces, resulting in the dominance of the (111) facet and the eventual formation of octahedral Cu_2_O. the octahedral Cu_2_O had eight (111) facets while cubic Cu_2_O had six (100) facets. The exposed facets of (111) for the octahedral and (100) for the cubic Cu_2_O, as confirmed by the lattice fringes in the HRTEM images, are shown in Figure 3. The lattice spacing was calculated to be 2.1 Å and 2.5 Å for the (100) and (111) facets, respectively. The synthesis of cubic Cu_2_O was similar to that of octahedral Cu_2_O, except for ascorbic acid being used as the reducing agent instead of hydrazine hydrate. Bai et al. reported that the addition of ascorbic acid led to the ionization of Cu^2+^ [32]. Vivas et al. prepared Cu_2_O particles, and they controlled the morphology by adjusting the NaOH concentration [33]. The morphology of the nanoparticles is determined by the lowest growth rate of the crystal faces, which, in turn, is influenced by the ratio between growth rates for different crystallographic directions [34].

### 3.2. Oxidation Stability of Cu_2_O in Humid Conditions

After two weeks of exposure to humid conditions, SEM images of Cu_2_O NPs showed no significant morphological changes. However, after four weeks, noticeable roughness and morphological changes were observed. After eight weeks of exposure to humid conditions, an increase in the particle size and the occurrence of agglomeration were observed. In order to investigate the comprehensive characteristics of both the bulk and surface, we conducted XRD and XPS analyses.

**Figure 4:**
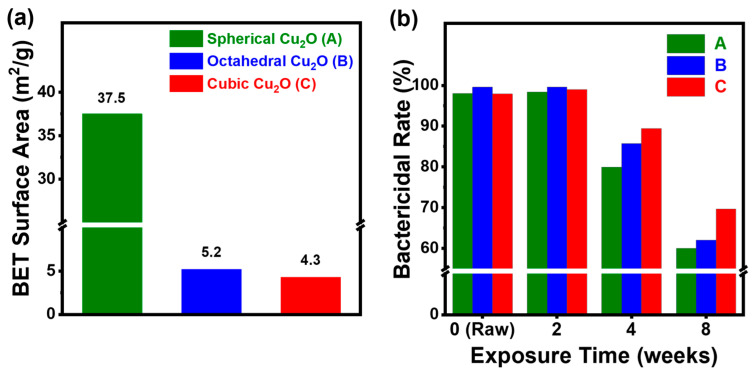
(**a**) BET specific surface area of Cu_2_O NPs obtained from conditions of not being exposed to humid conditions and (**b**) bactericidal rate (%) of Cu_2_O NPs with different morphologies exposed to humid conditions for 0 (raw), 2, 4, and 8 weeks.

The morphology and specific surface area influence the antimicrobial activity. In general, large surfaces result in more interaction with microorganisms, resulting in higher antimicrobial activity [35]. However, a surface area can increase the oxidation rate as the compound reacts with oxygen in the air, leading to a nonproportional relationship between the oxidation stability and the antimicrobial activity. Therefore, achieving a trade-off between surface area and oxidation stability is crucial for enhancing the antimicrobial activity of NPs. The BET specific surface areas of the spherical, octahedral, and cubic Cu_2_O were found to be 37.5, 5.2, and 4.3 m^2^/g, respectively (Figure 4a). The fact that spherical Cu_2_O had the largest surface area could be attributed to its porous structure. (Supplementary Materials, Figures S3 and S4) Furthermore, despite the octahedral and cubic Cu_2_O having smaller surface areas than spherical Cu_2_O, their antimicrobial activity was comparable to that of spherical Cu_2_O. Up to two weeks of exposure, the bactericidal rate of spherical, octahedral, and cubic Cu_2_O exceeded 98%. However, starting at four weeks, the bactericidal rate gradually decreased to 80%, 85%, and 89% for the three types of Cu_2_O, respectively. After eight weeks, their antimicrobial efficacy further decreased to 60%, 62%, and 70% (Figure 4b).

The difference in crystallinity and the ratio between the integrated intensities of the (111)/(100) facets suggest that raw samples have distinct crystal structures and morphologies. In order to determine the integrated intensity ratio of (111)/(100), we used Origin software and used a Gaussian function to analyze the XRD peak positions. For the raw samples, the ratio of the (111)/(100) facets for spherical, octahedral, and cubic Cu_2_O was calculated to be 3.46, 2.85, and 2.66, respectively. Furthermore, using the Debye–Scherrer formula, we predicted the crystal size of spherical, octahedral, and cubic Cu_2_O to be 6, 23, and 37 nm, respectively. The (111) facets, characterized by alternating layers of Cu^2+^ and O^2−^ on exposed surface of (111) facet, were highly reactive and readily interacted with microorganisms. By contrast, the (100) facet featured an O^2−^ terminated structure. Li et al. found that the (111) facets exhibited higher chemical reactivity than the (100) facets [36]. They attributed this difference to the presence of saturated chemical bonds on the (100) facets, which lacked dangling bonds [36]. Consequently, the (111) facets show higher adsorption and antimicrobial activity than the (100) facets [36]. The higher antimicrobial activity of the (111) facets has also been attributed to the effective generation and release of toxic copper ions [24]. The spherical Cu_2_O exhibited broad XRD patterns and the highest full width at half-maximum (FWHM) values for the (111) facets, which indicated its lower crystallinity compared with the other morphologies. Among the 0W samples, octahedral Cu_2_O exhibited a higher ratio of (111)/(100) facets compared with cubic Cu_2_O. Therefore, the difference in antimicrobial activity is attributed to the (111)/(100) ratio difference, and octahedral Cu_2_O was found to have a higher bactericidal rate than cubic Cu_2_O.

**Figure 5:**
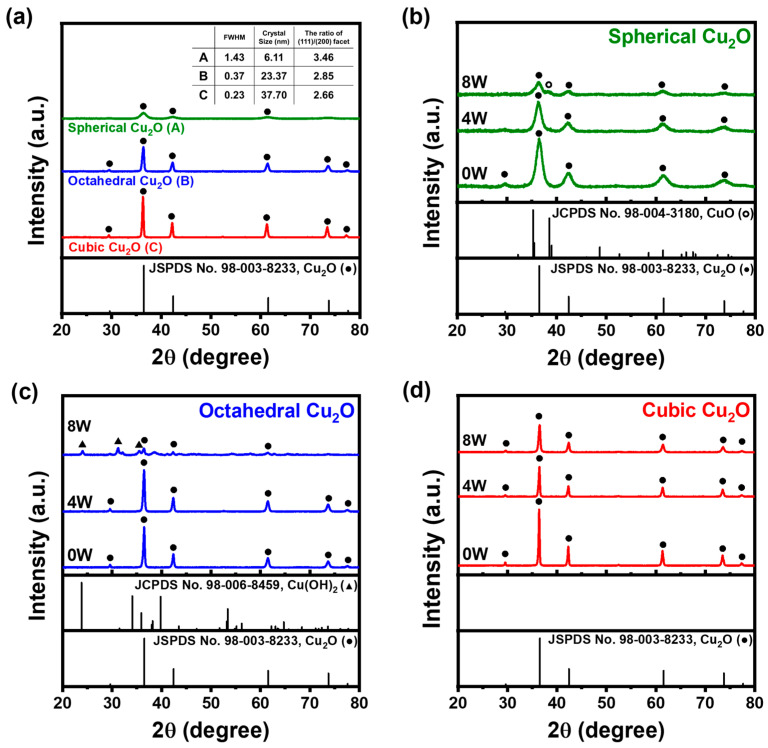
XRD patterns of Cu_2_O NPs: (**a**) 0W samples (●: Cu_2_O) and (**b**) spherical Cu_2_O, (**c**) octahedral Cu_2_O, and (**d**) cubic Cu_2_O exposed to humid conditions for 0, 4, and 8 weeks (●: Cu_2_O; ○: CuO; ▲: Cu(OH)_2_).

Figure 5a shows typical XRD patterns of Cu_2_O NPs with three different morphologies at zero, four, and eight weeks. After four weeks of exposure, peaks corresponding to only Cu_2_O (JCPDS 98-003-8233), namely, at 2*θ* = 29.65, 36.52, 42.42, 61.55, and 73.74, were observed for the raw samples. After eight weeks, spherical Cu_2_O showed the presence of CuO (JCPDS 98-004-3180); Cu_2_O patterns and octahedral Cu_2_O exhibited Cu(OH)_2_ (JCPDS 98-006-8456) and Cu_2_O patterns. However, the patterns of cubic Cu_2_O remained unchanged. A partial oxidation process resulted in the presence of both Cu^+^ and Cu^2+^ [37].

### 3.3. Surface Oxidation of Cu_2_O in Humid Conditions

**Figure 6:**
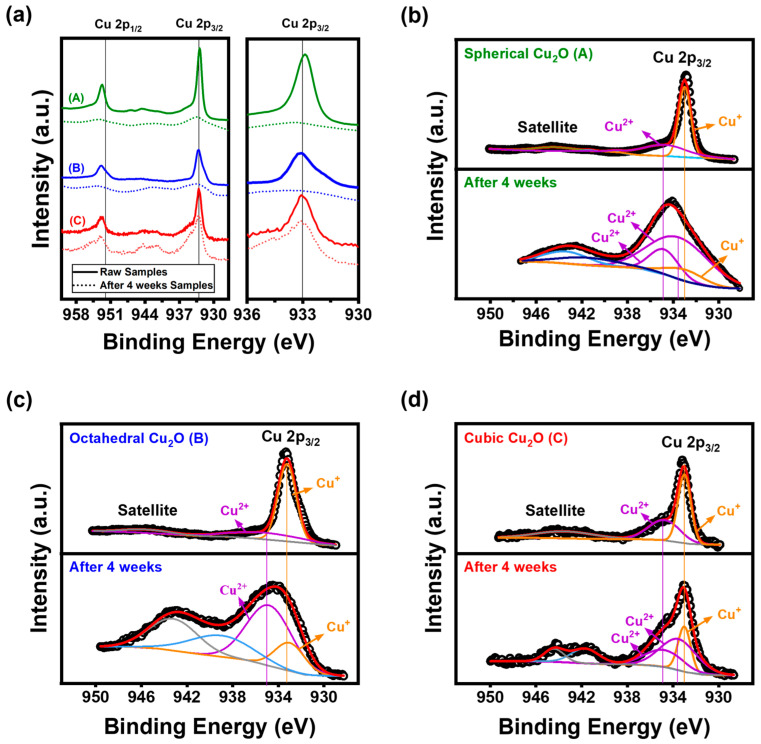
(**a**) Cu 2p region of the XPS spectra of samples and high-resolution XPS spectrum of the Cu 2p peak: (**b**) spherical Cu_2_O, (**c**) octahedral Cu_2_O, and (**d**) cubic Cu_2_O in humid conditions for zero and four weeks; (A: Spherical Cu_2_O, B: Octahedral Cu_2_O, C: Cubic Cu_2_O).

Despite the presence of only Cu_2_O peaks in the XRD patterns after four weeks (Figure 5), the bactericidal rate decreased (Figure 4b) and surface changes were observed (Figure 2). To investigate surface changes, we conducted an XPS analysis of the as-synthesized Cu_2_O NPs. The analysis indicated the oxidation states of the elements, as shown in Figure 6. The main peak of Cu_2_O at 933.0 eV is accompanied by a satellite peak in the range of 938 to 950 eV [38,39]. Other peaks centered at 933.5 and 934.8 eV correspond to the binding energy of CuO [40,41,42] and Cu(OH)^2^ [43]. The O 1s spectra of samples in Figure S5 have peak values of 530.1, 530.9, and 533.5 eV, which were assigned the lattice oxygen in Cu_2_O, adsorbed O_2_, and O-H bond, respectively [44,45].

For the spherical and octahedral Cu_2_O samples exposed for four weeks, the overlapping satellite peak observed in the results confirms the transformation of Cu^+^ to Cu^2+^. The overlapping satellite peak observed in the spectrum indicates the presence of impurities, and these impurities are likely the cause of the distortion in the main peak that leads to a broader and less defined morphology. The FWHM of the Cu^+^ peaks for spherical, octahedral, and cubic Cu_2_O changed from 0.62, 1.72, and 1.30 at zero weeks to 7.30, 3.05, and 1.35 at four weeks, respectively. Cubic Cu_2_O exhibited fewer compositional changes on its surface compared with octahedral and spherical Cu_2_O. Furthermore, the binding energy shifted toward higher values, indicating the formation of Cu_2_O/CuO and Cu_2_O/Cu(OH)_2_ heterojunctions. The XPS analysis confirmed the coexistence of different oxidation states of copper in the synthesized Cu_2_O NPs.

In order to quantify the amount of Cu^+^ and Cu^2+^, we calculated the ratio between the peak areas of Cu^2+^ and Cu^+^ in the Cu 2p_2/3_ absorption spectra for different degrees of oxidation [46,47]. For the raw samples, the calculated values of this ratio for spherical, octahedral, and cubic Cu_2_O were 0.62, 0.33, 0.73, respectively, and the values after sample exposure for four weeks were 7.30, 3.18, and 3.13. Spherical Cu_2_O is more easily oxidized owing to its large surface area. Furthermore, the relative oxidation stability of Cu_2_O(100) is higher than the (111) facets because of the lower surface energy of the (100) facets [48]. The significant increase in the calculated peak area ratio after exposure for four weeks indicated a higher proportion of Cu^2+^ in the samples, suggesting that more Cu^+^ was transformed to Cu^2+^. Also, the peak area of four weeks for Cu_2_O samples corresponding to the O-H binding energy in octahedral Cu_2_O is broader than that in cubic Cu_2_O, as shown in Figure S1. Some Cu^2+^ peaks were observed for the raw samples because of the presence of NaOH in the sample, introduced during sample preparation.

After exposure for four weeks, the XPS analysis revealed the presence of Cu(OH)_2_ and CuO peaks for the surfaces of spherical and cubic Cu_2_O, whereas only Cu(OH)_2_ peaks were observed for the surface of octahedral Cu_2_O. These results can be attributed to H_2_O dissociation on the Cu_2_O surface, which has been reported to depend on the surface crystallographic orientation. The mechanism of H_2_O dissociation is as follows: (1) H_2_O (g) → H_2_O (ads), (2) H_2_O (ads) → H (ads) + OH (ads), and (3) OH (ads) → H (ads) + O (ads) [49]. According to XPS results from Schulz and Cox’s study, the [Cu]/[O] ratio follows the order of (111) > (100) [50]. This indicates that the (111) surface tends to be Cu-terminated, while the (100) surface is O-terminated. On the Cu_2_O(111) facets, Cu predominantly adsorbs OH^−^, forming a Cu(OH)_2_ structure. Subsequently, weak hydrogen bonds (O-H) break, leading to the structure’s transformation into CuO. Copper hydroxide (Cu(OH)_2_) is a metastable phase that readily transforms into the more stable CuO [51]. These results indicate that the ratio between the peak areas of Cu^2+^ and Cu^+^ species is higher for octahedral Cu_2_O compared with cubic Cu_2_O.

### 3.4. Oxidation Stability of Cu_2_O in Thermal Conditions

**Figure 7:**
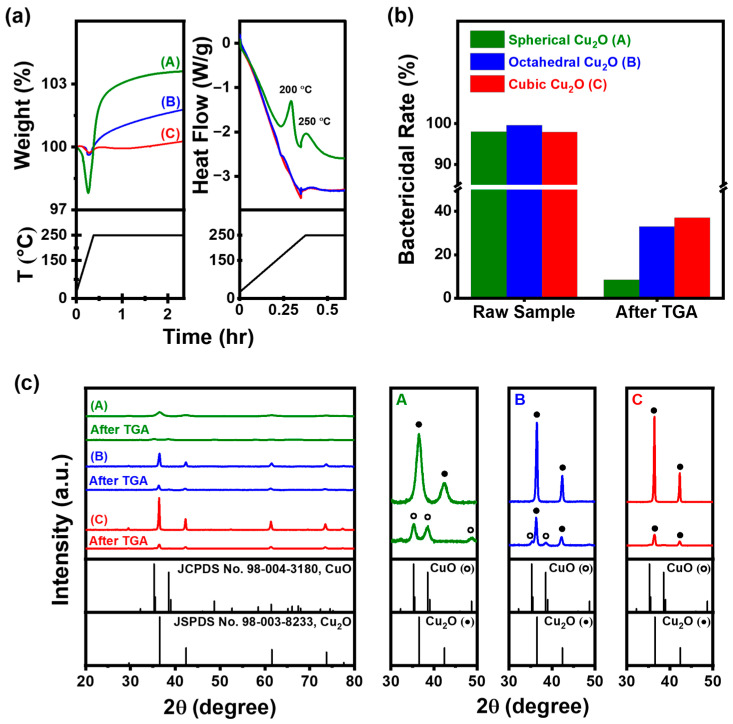
(**a**) TGA-DSC curves at 250 °C for 2 h, (**b**) bactericidal rate of Cu_2_O NPs in raw samples and following TGA, and (**c**) XRD patterns of Cu_2_O NPs (●: Cu_2_O; ○: CuO); (A: Spherical Cu_2_O, B: Octahedral Cu_2_O, C: Cubic Cu_2_O).

TGA and DSC curves of Cu_2_O NPs with various morphologies are presented in Figure 7a for the temperature range of 25–250 °C. TGA was employed to determine the weight percentage (wt%) of Cu_2_O NPs in the produced Cu_2_O. Water and organics that had been adsorbed on the surface of the samples evaporated, resulting in an initial weight loss of approximately 2.2 wt% for spherical Cu_2_O and 0.3 wt% for octahedral and cubic Cu_2_O. The weight of Cu_2_O increased from 166 °C in spherical Cu_2_O and from 195 °C in octahedral and cubic Cu_2_O owing to the oxidation of Cu_2_O to CuO. Further investigation into the rate of weight gain revealed that it was higher for spherical Cu_2_O compared with octahedral and cubic Cu_2_O, both of which showed a more gradual weight gain. This observation suggests that the oxidation rate of spherical Cu_2_O was higher than that of octahedral and cubic Cu_2_O. The final weight gain was 3.6%, 1.7%, and 0.26% for spherical, octahedral, and cubic Cu_2_O, respectively. The DSC curve in Figure 7a shows exothermic heat flow peaks around 200 and 250 °C, which are attributed to the oxidation of CuO and the decrease in the oxidation rate from the stage where a constant temperature was maintained, respectively.

Bactericidal rates of spherical, octahedral, and cubic Cu_2_O decreased from 98%, 99.59%, and 97.93% in raw samples to 8.5%, 32.9%, and 37% after TGA, respectively. XRD patterns of the samples showed a decrease in the XRD Bragg peak intensity of Cu_2_O NPs, which could be attributed to the thermal excitation of lattice vibrations. The lattice distortion caused by the displacement of neighboring atoms would have facilitated diffusion [52,53]. The temperature-dependent coefficient of oxygen surface diffusion could be described by an Arrhenius relationship. At high temperatures, the diffusion distance of oxygen on the Cu_2_O surface increases, enhancing the probability of oxygen atoms being captured by the oxide islands [53,54].

After TGA, XRD patterns showed that only CuO (JCPDS 98-004-3180) peaks were observed at 2*θ* = 35.26°, 38.54°, 48.70°, 58.51°, 61.40°, 66.39°, and 67.43° for spherical Cu_2_O, while both Cu_2_O and CuO peaks were observed for octahedral Cu_2_O. Cubic Cu_2_O exhibited a stable Cu_2_O peak. Spherical Cu_2_O, with low crystallinity and a large surface area, exhibited high oxidation. On the other hand, despite octahedral and cubic Cu_2_O having relatively similar surface areas and particle sizes, differences in their thermal effects were observed. These results indicate that the Cu_2_O(111) surface exhibits a higher reactivity and, hence, higher chemisorption capacity compared with the Cu_2_O(100) surface. This behavior can be attributed to the presence of Cu atoms on the Cu_2_O(111) surface [55].

## 4. Conclusions

Copper oxide (Cu_2_O) NPs with various morphologies (spherical, octahedral, and cubic morphologies) and facets were synthesized. In humid conditions (20 ± 5 °C, 85 ± 5% humidity), the adsorption of OH^−^ groups on the Cu_2_O surface led to the transformation of the compound to Cu(OH)_2_ and CuO, and both of these resulting compounds have lower antimicrobial activity than Cu_2_O. Among the three morphologies, cubic Cu_2_O with exposed (100) facets exhibited superior stability in humid and thermal conditions. Thus, the effect of the morphology and crystallinity of Cu_2_O NPs on their antimicrobial activity and oxidation stability is stronger than that of the specific surface area and particle size. Acquiring the capability to control the morphology of Cu_2_O and an understanding of its stability in oxidative environments can facilitate the use of the compound in a wide range of applications, such as in catalysis, sensors, and electrodes.

## Data Availability

Data are contained within the article and supplementary materials.

## Supplementary Materials

The following supporting information can be downloaded at: https://www.mdpi.com/article/10.3390/ma17010261/s1, Figure S1. SEM images of the octahedral Cu_2_O particles prepared at different temperatures. Figure S2. XRD patterns of the octahedral Cu_2_O particles prepared at different temperatures. Figure S3. Specific surface area of Cu_2_O particles with three distinct morphologies. Figure S4. SEM images of spherical Cu_2_O particles. Figure S5. High resolution XPS of the O 1s peak of octahedral and cubic Cu_2_O for zero and four weeks.
