# Supplementary material for: Antimicrobial Activity of Morphology-Controlled Cu2O Nanoparticles: Oxidation Stability under Humid and Thermal Conditions
Source: Materials (Basel). 2024 Jan 4;17(1):261. doi: 10.3390/ma17010261 (PMC10780029; doi:10.3390/ma17010261)
Supplement: Supplementary file 1 [file materials-17-00261-s001.zip › materials-2793708-supplementary.pdf]

materials

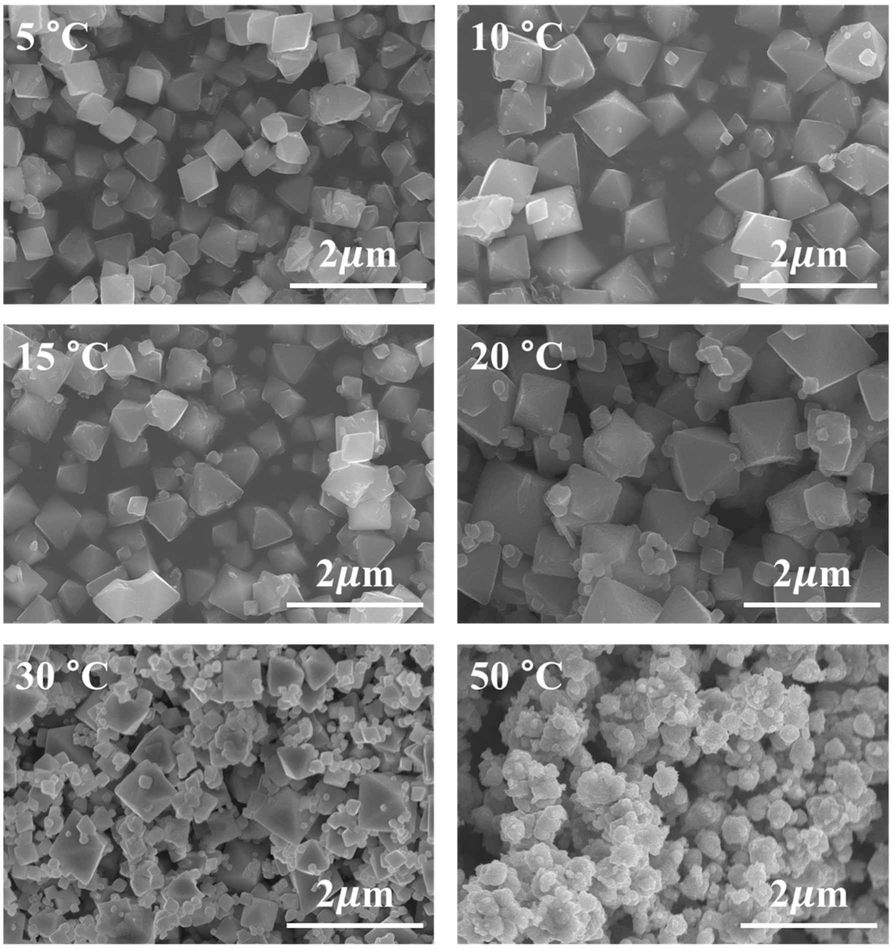

Figure S1. SEM images of the octahedral Cu_2O particles prepared at different temperatures.

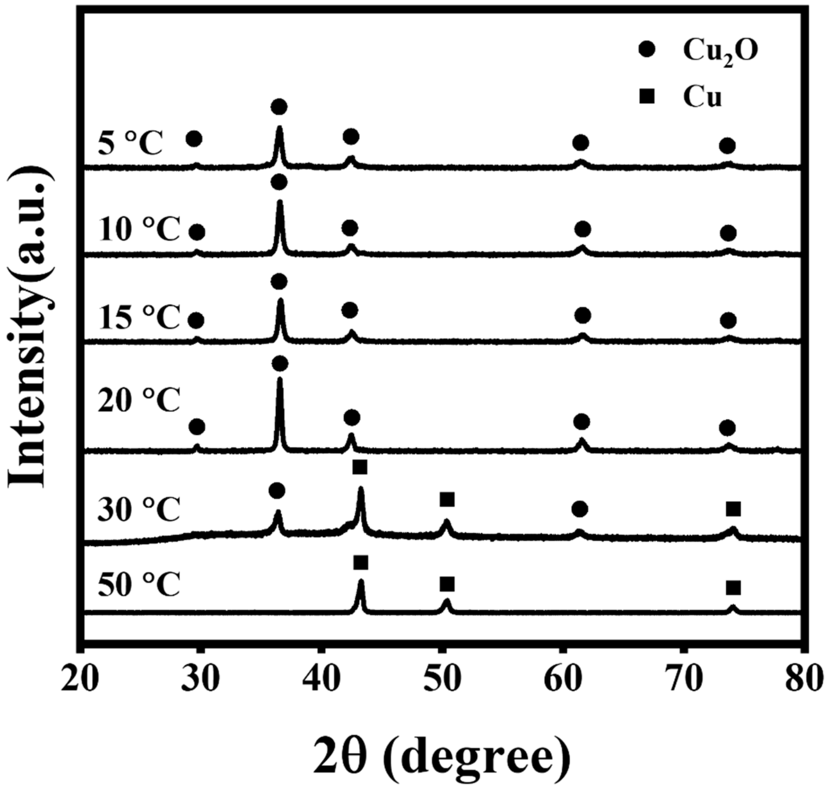

Figure S2. XRD patterns of the octahedral Cu_2O particles prepared at different temperatures.

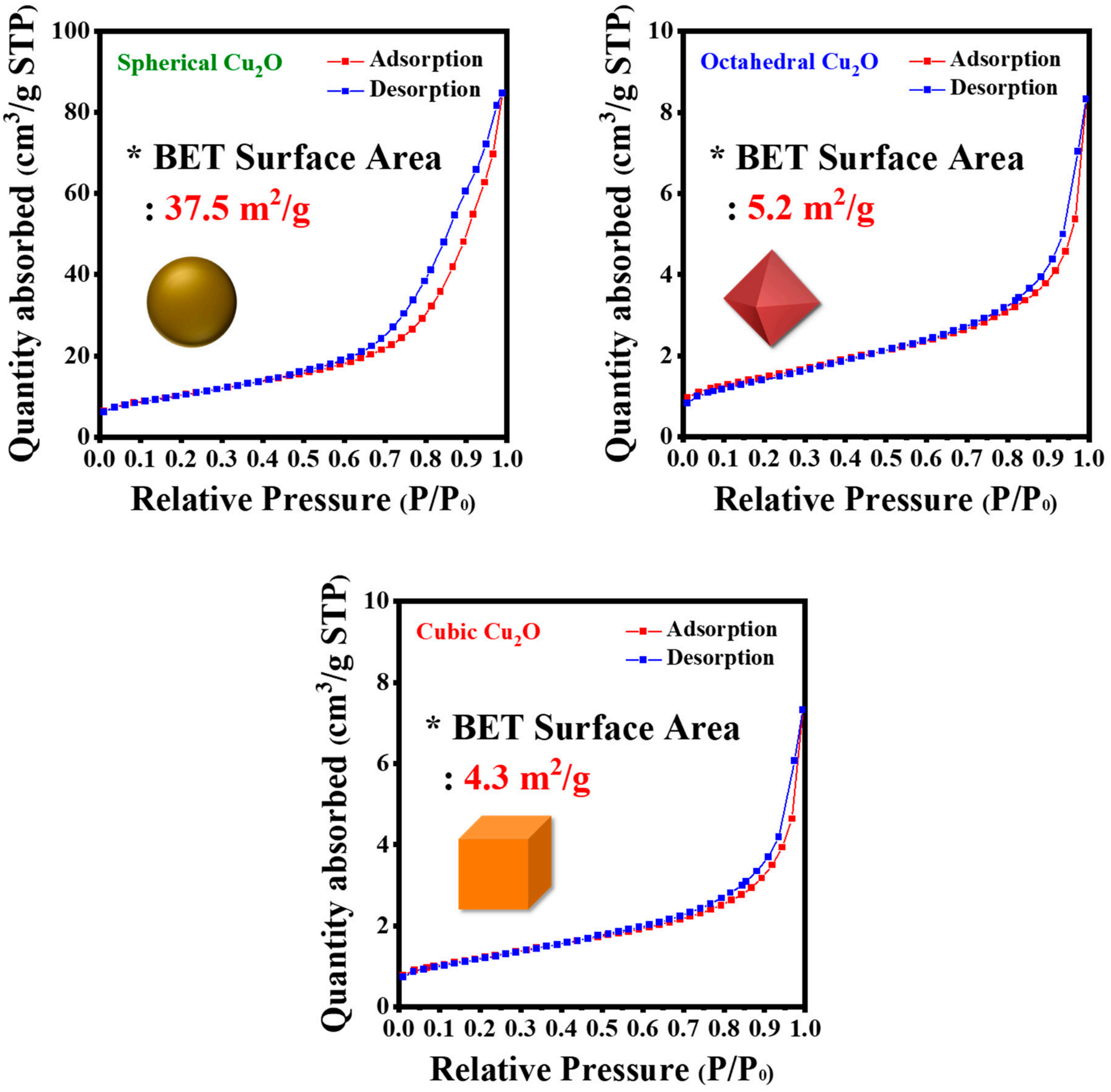

Figure S3. Specific surface area of Cu_2O particles with three distinct morphologies.

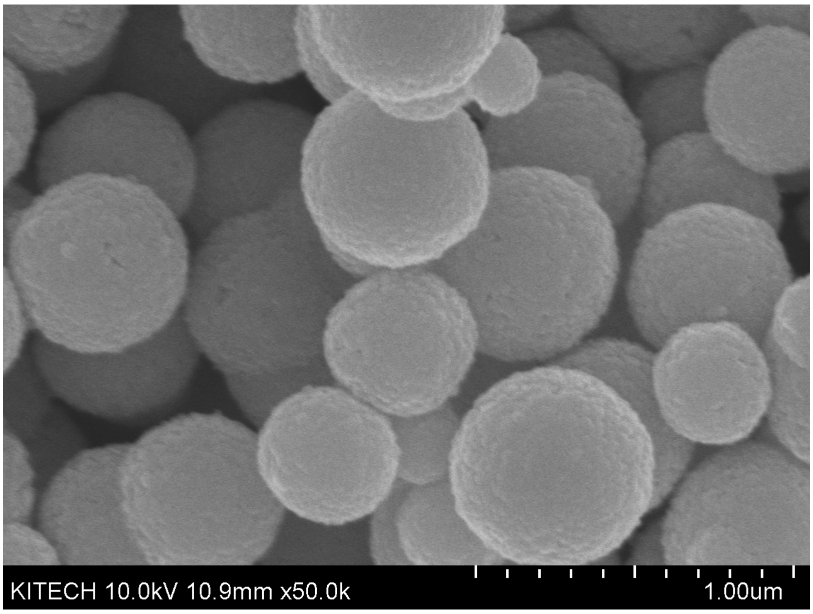

Figure S4. SEM images of spherical Cu_2O particles.

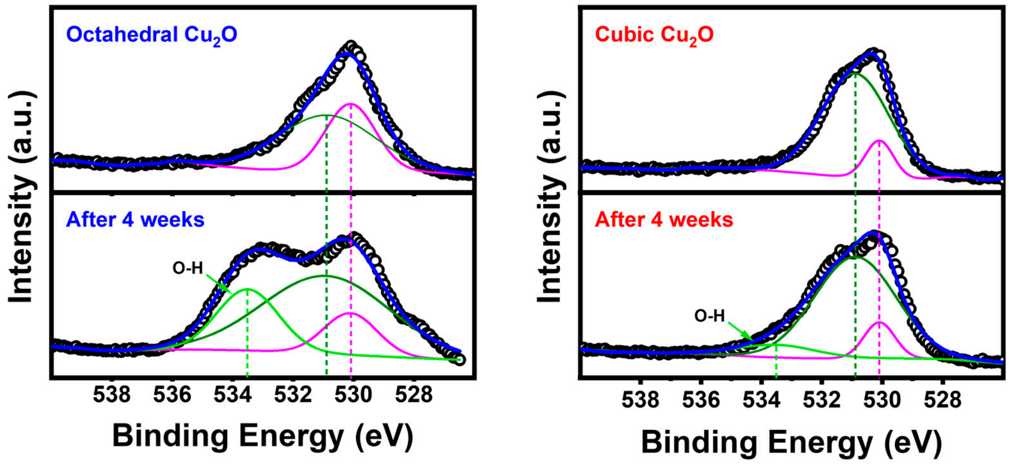

Figure S5. High resolution XPS of the O 1s peak of octahedral and cubic Cu_2O for zero and four weeks.
